# Use of Multiple-Choice Items in Summative Examinations: Questionnaire Survey Among German Undergraduate Dental Training Programs

**DOI:** 10.2196/58126

**Published:** 2024-06-27

**Authors:** Lena Rössler, Manfred Herrmann, Annette Wiegand, Philipp Kanzow

**Affiliations:** 1 Department of Preventive Dentistry, Periodontology and Cariology, University Medical Center Göttingen, Göttingen, Germany; 2 Study Deanery, University Medical Center Göttingen, Göttingen, Germany

**Keywords:** alternate-choice, assessment, best-answer, dental, dental schools, dental training, education, educational assessment, educational measurement, examination, German, Germany, k of n, Kprim, K’, medical education, medical student, MTF, Multiple-True-False, multiple choice, multiple-select, Pick-N, scoring, scoring system, single choice, single response, test, testing, true/false, true-false, Type A, Type K, Type K’, Type R, Type X, undergraduate, undergraduate curriculum, undergraduate education

## Abstract

**Background:**

Multiple-choice examinations are frequently used in German dental schools. However, details regarding the used item types and applied scoring methods are lacking.

**Objective:**

This study aims to gain insight into the current use of multiple-choice items (ie, questions) in summative examinations in German undergraduate dental training programs.

**Methods:**

A paper-based 10-item questionnaire regarding the used assessment methods, multiple-choice item types, and applied scoring methods was designed. The pilot-tested questionnaire was mailed to the deans of studies and to the heads of the Department of Operative/Restorative Dentistry at all 30 dental schools in Germany in February 2023. Statistical analysis was performed using the Fisher exact test (*P*<.05).

**Results:**

The response rate amounted to 90% (27/30 dental schools). All respondent dental schools used multiple-choice examinations for summative assessments. Examinations were delivered electronically by 70% (19/27) of the dental schools. Almost all dental schools used single-choice Type A items (24/27, 89%), which accounted for the largest number of items in approximately half of the dental schools (13/27, 48%). Further item types (eg, conventional multiple-select items, Multiple-True-False, and Pick-N) were only used by fewer dental schools (≤67%, up to 18 out of 27 dental schools). For the multiple-select item types, the applied scoring methods varied considerably (ie, awarding [intermediate] partial credit and requirements for partial credit). Dental schools with the possibility of electronic examinations used multiple-select items slightly more often (14/19, 74% vs 4/8, 50%). However, this difference was statistically not significant (*P*=.38). Dental schools used items either individually or as key feature problems consisting of a clinical case scenario followed by a number of items focusing on critical treatment steps (15/27, 56%). Not a single school used alternative testing methods (eg, answer-until-correct). A formal item review process was established at about half of the dental schools (15/27, 56%).

**Conclusions:**

Summative assessment methods among German dental schools vary widely. Especially, a large variability regarding the use and scoring of multiple-select multiple-choice items was found.

## Introduction

Summative examinations of theoretical knowledge are an integral part of university degree programs. As they are intended to assess examinees’ ability regarding predefined learning objectives, they should reflect examinees’ true knowledge as closely as possible. To assess examinees objectively and efficiently, multiple-choice examinations were described as early as 1916 [1,2]. To date, these types of examinations have been expanded by further item types, and multiple-choice examinations are frequently used within higher education including but not limited to dental training programs [3-5]. Multiple-choice items (ie, questions) can be subdivided into single-choice items (eg, Type A, Type K, Type R, and alternate-choice) and multiple-select items (eg, Pick-N and Multiple-True-False [Type K’]) [6]. While dichotomous scoring (ie, 1 full credit point is awarded if examinees mark the correct answer option or statements, otherwise no credit is awarded) is most commonly proposed for single-choice items [7], scoring methods for multiple-select items are more heterogeneous: Besides dichotomous scoring, further scoring methods resulting in (intermediate) partial credit or even negative points (ie, malus points) have been described [8,9].

Besides paper-based examinations, examinations are nowadays frequently delivered electronically. While electronic examinations are well perceived by examinees [10], comprehensive studies regarding their effectiveness are still lacking [11]. However, the use of different examination software (eg, UCAN’s [Umbrella Consortium for Assessment Networks] CAMPUS examination software) might improve the ease of multiple-choice examinations, accelerate the evaluation of examinations and item analysis, and allow for more complex scoring algorithms. Despite the benefits associated with electronic examinations, the availability of hardware and software at the level of individual institutions might limit its use.

In Germany, the revised undergraduate dental curriculum consists of 10 semesters and includes preclinical training (4 semesters), training using simulators or phantom heads (2 semesters), and clinical training (4 semesters). Following the state examinations after each part (ie, after the fourth, sixth, and 10th semester), students receive their license (“Approbation”) to practice dentistry. Besides practical skills, theoretical knowledge is taught within the undergraduate dental curriculum, and students’ ability is often assessed using written multiple-choice examinations. However, such examinations are not standardized among German dental schools. While general recommendations exist for their design and evaluation [12,13], details such as suitable item types and applied scoring methods are often defined in local examination guidelines at the level of individual dental schools. However, these details might impact examinees’ scoring results [5]. To the best of our knowledge, a comprehensive overview regarding the used item types and applied scoring methods at German dental schools does not exist.

Therefore, this study aimed to gain insight into the current use of multiple-choice items in summative examinations in German undergraduate dental training programs. The null hypothesis is that the use of digital examinations does not impact the use of more complex (ie, multiple-select) multiple-choice items.

## Methods

### Ethical Considerations

The study was designed as a prospective investigation. In preparation for the investigation, the websites of all German dental schools were screened (n=30), and the names of the heads of the Department of Operative/Restorative Dentistry and the deans of studies were noted for later procedures.

The study was performed after approval by the local ethics committee of the University Medical Center Göttingen (approval number 22/1/23). Participation in this study was voluntary, and participants gave their informed consent for the anonymous evaluation of the provided answers by returning the questionnaires. Participants did not receive any incentives or compensation.

### Questionnaire

A paper-based questionnaire, consisting of 10 items about the construction and evaluation of summative examinations, was jointly designed by the authors and pilot-tested in the University Medical Center Göttingen (Multimedia Appendix 1). Both closed and open-ended items were used. The opening questions related to different examination types used for the summative assessment of theoretical knowledge, and whether or not electronic examinations were being used. Additionally, it was asked whether the examination items undergo a formal review process and if so, the participants had the chance to give a brief description of this procedure. The more specific questions related to the types of multiple-choice items used and asked for the relative percentage to which these items were being used. Furthermore, the participants were asked to describe the applied scoring methods for each of the item types used. Finally, participants were provided with a text field open for comments and their contact details (ie, if required for further clarification) and were asked to supply a copy of their local examination guidelines or program regulations.

**Figure 1.**
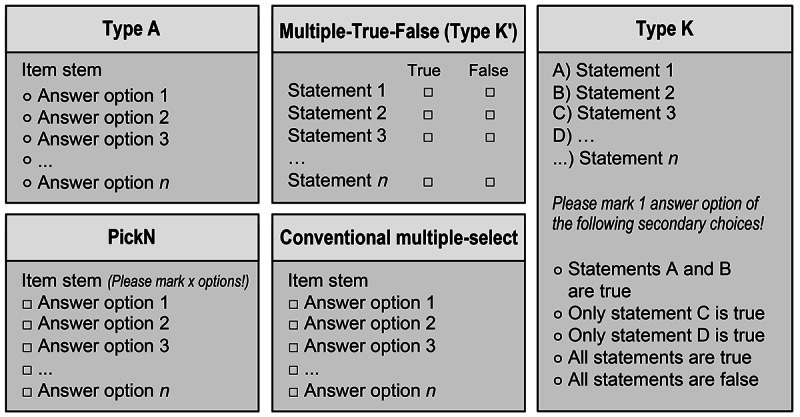
Exemplary presentation of the most commonly used multiple-choice item types referenced in the questionnaire. Round marking boxes represent 1 answer option to be selected (1 out of x), while square marking boxes imply that multiple answer options or statements (x out of X) can be chosen.

Following the evaluation of the pilot survey among 5 dentists at the University Medical Center Göttingen, the questionnaire was slightly modified for clarification, printed, and mailed to (1) the heads of the Department of Operative/Restorative Dentistry and to (2) the deans of studies on February 1, 2023. The wording was slightly adjusted for each recipient: (1) “used in your department” versus (2) “permitted at your dental school”. Mailings included a personalized cover letter, an overview illustrating different multiple-choice item types (Figure 1), and a stamped return envelope. The survey was closed after 12 weeks. Nonresponders were reminded once 6 weeks after the initial distribution of the questionnaires.

### Statistical Analysis

First, data were manually transferred into a digital chart using a piloted spreadsheet containing columns for each item of the questionnaire. This step was independently performed by 2 authors (LR and PK). In case of disagreement, data were repeatedly extracted from the returned questionnaires.

In case of disagreement between the heads of the Department of Operative/Restorative Dentistry and the deans of studies, results were based on the responses from the heads of the Department of Operative/Restorative Dentistry. For further clarification, responses were cross-validated with the supplied or publicly available examination guidelines and program regulations. If required, respondents were contacted for further clarification if they had agreed to do so previously.

Second, statistical analysis was performed using the software SPSS Statistics (Macintosh version 29.0.0.0; IBM Corp). The effect of delivering digital examinations on the use of multiple-select items was assessed using the Fisher exact test. The level of significance was set at .05.

## Results

### Overview

In total, responses from 27 dental schools were received yielding a response rate of 90% (27/30 dental schools). More specifically, 25 Departments of Operative/Restorative Dentistry and 17 deans of studies replied. All dental schools responded that they use written multiple-choice examinations for the assessment of examinees’ theoretical knowledge. Therefore, subsequent results are based on the number of respondent dental schools.

### Multiple-Choice Items Used

The most commonly used multiple-choice item types at German dental schools were single-choice Type A or Type A_negative_ items with 3 to 6 answer options (24/27, 89%). Pick-N items (ie, the number of answer options to be selected is known to examinees) were reported to contain between 3 and 26 answer options and were used by 67% (18/27) of dental schools. Type K items were reported to contain between 3 and 6 statements and were used by 52% (14/27) of the dental schools. Multiple-True-False (also known under further names such as Kprim, Type K’, or Type X) and conventional multiple-select items (ie, the number of answer options to be selected is unknown to examinees) were reported to contain between 4 and 6 statements or answer options and were both used by 44% (12/27) of the dental schools. The use of further item types is shown in Table 1.

**Table 1.** Different multiple-choice item types for the assessment of theoretical knowledge at the respondent dental schools (N=27).

Item type	Dental schools, n (%)
Type A	24 (89)
Pick-N	18 (67)
Type K	14 (52)
Conventional multiple-select	12 (44)
Multiple-True-False (Type K’)	12 (44)
Type R	6 (22)
Alternate-choice	4 (15)

### Examination Setting

Key feature problems consisting of a clinical case scenario followed by a number of items focusing on critical treatment steps were used by approximately half of the dental schools (15/27, 56%). Not a single school used alternative testing methods (eg, answer-until-correct). Also, a formal item review process prior to the delivery of the examination was only established at about half of the dental schools (15/27, 56%).

### Delivery of Examinations

The percentage of dental schools that deliver examinations electronically amounted to 70% (19/27). However, the software used by the dental schools differed: a dedicated examination software (ie, UCAN’s CAMPUS or tEXAM, Q-Exam [IQUL GmbH]) was used by 8 dental schools, while learning management systems such as Moodle (Moodle Pty Ltd), ILIAS (ILIAS open source e-Learning e.V.), or OpenOLAT (frentix GmbH) were used by 7 dental schools for the purpose of examination delivery. The remaining 4 dental schools did not provide any information regarding the examination software they used.

Dental schools with the possibility of electronic examinations used multiple-select items slightly more often (14/19, 74% vs 4/8, 50%). However, this difference was statistically not significant (*P*=.38).

### Applied Scoring Methods

All dental schools scored single-choice items (ie, Type A, Type A_negative_, Type K, Type R, and alternate-choice) dichotomously (ie, 1 full credit point is awarded if examinees mark the correct answer option or statements, otherwise no credit is awarded).

Scoring of multiple-select items was more heterogeneous and no single scoring method that was commonly used was identified: some dental schools used scoring algorithms resulting in partial (ie, 0.5 credit points) or intermediate partial credit (ie, 1/n partial credit for each correct response) besides dichotomous scoring on multiple-select items. However, scoring methods resulting in negative points (ie, malus points) were not used at any location.

## Discussion

### Principal Findings

The aim of this study was to gain insight into summative assessment methods that involve the use of multiple-choice items and are used at German dental schools. The purpose of summative assessment is to evaluate examinees’ knowledge at the end of a course by comparing their scores to a predefined standard (ie, cutoff score) [14]. Our results demonstrate that all respondent dental schools use multiple-choice examinations for summative assessment of theoretical knowledge. Besides individual items, approximately half of the dental schools also use key feature problems.

Single-choice Type A items are the most popular item types used at German dental schools. These items are used by almost every respondent dental school and often account for the largest number of items at the respective dental schools. This might be explained by the demand for ease of scoring (ie, dichotomous scoring, no partially correct responses).

Multiple-select item types such as Pick-N or Multiple-True-False are used by fewer dental schools. For these item types, the applied scoring methods vary considerably: Some dental schools award partial or even intermediate partial credit for partially correct responses while others do not. However, the exact cutoff levels and scoring methods for partial credit differed. For example, Partial Scoring 50% (PS_50_) was used by some dental schools for Pick-N items: In these cases, 1 full credit point is awarded if all answer options are marked correctly, and 0.5 credit points are awarded if at least half of the true answer options are marked, otherwise no credit is awarded [9,15]. Furthermore, a similar scoring method named Half-point Scoring was used by some dental schools for Multiple-True-False and conventional multiple-select items: 1 full credit point is awarded if all statements or answer options are marked correctly, 0.5 credit points are awarded if the response to 1 statement or answer option is incorrect, otherwise no credit is awarded [8,16]. In addition, some dental schools awarded intermediate partial credit on multiple-select items: In the case of Partial Scoring 1/n (PS_1/n_), 1/n partial credit was awarded for each correct response [8,9]. Some dental schools also subtracted 1/n partial credit for each incorrect response (Blasberg-Method) [8,9,17].

As a result, the scoring of multiple-select items at different German dental schools can be considered very heterogeneous. This is not surprising, as a vast number of different scoring methods for multiple-select items have been described in the literature [8,9]. As stated previously, it is not possible to suggest a single versatile scoring method. Different requirements as defined in dental schools’ local examination guidelines (eg, fixed pass-mark and fixed proportion of true answer options) impact the scoring method to be selected. Regarding jurisdictional requirements, scoring methods resulting in negative points (ie, malus points) must not be used in Germany [13]. Consequently, not a single dental school uses scoring methods resulting in malus points. However, almost half of the dental schools do not use a formal item review process. A formal review process is recommended prior to the delivery of the examinations and might further improve the quality and overall validity of the examinations.

In addition, 70% (19/27) of all dental schools stated to deliver examinations electronically. While the electronic delivery of examinations allows for automatic scoring and more complex scoring methods (ie, within the context of multiple-select items), no statistically significant relation between the type of delivery (paper-based vs electronic) and the use of multiple-select item types was found. Therefore, our results fail to reject the null hypothesis. This might be explained by the software used for the delivery and scoring of electronic examinations: it was found that dental schools use learning management systems such as Moodle, ILIAS, or OpenOLAT besides dedicated examination software such as UCAN’s CAMPUS, UCAN’s tEXAM, or Q-Exam for the delivery and scoring of summative assessments. This is of relevance, as learning management systems usually support fewer item types and scoring methods than dedicated examination software [8,9]. As a result, electronic delivery of examinations does not necessarily result in an increased use of multiple-select items.

Interestingly, not a single dental school used alternative testing methods that deviate from the standard setting during examinations (ie, examinees mark the answer options or statements they believe to be correct or true but receive no immediate feedback regarding correctly or incorrectly marked answer options or statements). Within multiple-choice examinations, alternative testing methods such as confidence weighting scoring (ie, examinees are requested to indicate the degree of confidence in their marking) [18], elimination scoring (ie, examinees are instructed to mark the incorrect instead of correct answer options) [19], or answer-until-correct [20,21] have been described in the literature. Within the answer-until-correct method, examinees receive immediate feedback and examinees may correct their marking on previously incorrectly marked items, thereby still receiving partial credit. However, the benefit of such testing methods within the field of dental education is questionable. Dental school examinees are becoming future dentists. While treating patients, dentists are required to make informed choices and dentists might not always have a second chance without potentially harming their patients. In addition, such alternative testing methods benefit from the electronic delivery of examinations and set even higher requirements for the used examination software.

### Strengths and Limitations

To the best of our knowledge, this is the first study to systematically assess the use and scoring of multiple-choice item types in summative examinations among German dental schools. A number of strengths are present. First, a pretested questionnaire was used. Second, our questionnaire survey study yielded a high response rate of 90% (27/30 dental schools). Third, our results might be considered representative of the current use of multiple-choice items in summative examinations among German dental schools.

Nevertheless, limitations are also present. First, our questionnaire focused on multiple-choice items; therefore, the use of other assessment types (eg, objective structured clinical examinations, oral examinations) remains unknown. Second, the number of dental schools in Germany is limited. Thereby, results from the Fisher exact test might be underpowered despite the high response rate. Furthermore, this study could not control for potential confounders (eg, location, number of students per dental school) due to the overall low number of dental schools. Third, transferability and generalizability to other educational settings might be limited due to different jurisdictional requirements or the overall lower importance of written examinations.

### Future Directions

New dental licensing regulations (“Approbationsordnung”) have been in effect since 2021, which restructured the undergraduate dental curriculum in Germany. For the first time, a nationwide written board examination with single-choice items takes place at the end of all undergraduate dental programs (ie, after the 10th semester) [22]. Therefore, multiple-choice examinations in general and especially single-choice Type A items will remain a popular format for summative examinations among German undergraduate dental programs. Ideally, examinees already become familiar with single-choice Type A items during their studies. Therefore, all dental schools should use single-choice Type A items to adequately prepare their students for the final board examination.

Nevertheless, additional examinations (eg, objective structured clinical or practical examinations) are required to test examinees’ practical skills [3]. Regardless of the used item type, multiple-choice examinations are not suitable to assess the higher levels Miller’s Pyramid of clinical competence (ie, does and shows how) [23].

### Conclusion

While students from almost all dental schools can be expected to be familiar with single-choice Type A items, techniques for the summative assessment of theoretical knowledge differ widely among German dental schools. Especially, a large variability regarding the use and scoring of multiple-select multiple-choice items was found. In addition, implementing a formal item review process might further improve the quality and overall validity of the examinations.

## Supplementary material

10.2196/58126 Multimedia Appendix 1 Authors’ translation of the used questionnaire, which was originally distributed in German.

## Abbreviations

PS_1/n_: Partial Scoring 1/n
PS_50_: Partial Scoring 50%
UCAN: Umbrella Consortium for Assessment Networks
