# Supplementary material for: Use of Multiple-Choice Items in Summative Examinations: Questionnaire Survey Among German Undergraduate Dental Training Programs
Source: JMIR Med Educ. 2024 Jun 27;10:e58126. doi: 10.2196/58126 (PMC11220727; doi:10.2196/58126)
Supplement: Multimedia Appendix 1 [file mededu-v10-e58126-s001.pdf]

Questionnaire Survey Regarding the Use and Scoring of Multiple-Choice Items in Summative Examinations in Undergraduate Dental Training Programs

1. Please provide the name of your dental school.

____

2. Which examination types are used in your department / are permitted at your dental school to assess students' theoretical knowledge in summative examinations (examinations that must be passed to achieve the course objective)? (*multiple selections possible*)

	Used in department	Permitted at dental school *
Presentation	☐	☐
Oral examination	☐	☐
Structured oral examination (SOE)	☐	☐
Written paper (eg, term paper, seminar paper, project work, protocol, portfolio)	☐	☐
Written examination (electronic)	☐	☐
Written examination (paper-based)	☐	☐
Other: ________________________	☐	☐

* according to your examination and program regulations, local examination guidelines, etc

3. Which item types are used in your department / are permitted at your dental school for written examinations? (*multiple selections possible*)

	Used in department	Permitted at dental school *
Open items (essay / short answer items)	☐	☐
Fill-in-the-blank / text subset items	☐	☐
Spot the mistake items	☐	☐
Interval / numerical items	☐	☐
Classification / sequencing / grouping items	☐	☐
Long-menu items	☐	☐
Picture analysis items	☐	☐
Multiple-choice (single-choice / multiple-select items)	☐	☐
Other: ________________________	☐	☐

* according to your examination and program regulations, local examination guidelines, etc

4. Are items used individually or in form of cases with follow-up items (eg, key-feature problems) in your department / at your dental school?

	Used in department	Permitted at dental school *
Key-feature problems	☐	☐
Modified essay questions (MEQ)	☐	☐
Script concordance test (SCT)	☐	☐
Other: ________________________	☐	☐

* according to your examination and program regulations, local examination guidelines, etc

Please turn →

5. If examinations are delivered electronically in your department / at your dental school, which examination software is used?

____

6. Which multiple-choice item types (please see attached schematic overview) are used in your department / are permitted at your dental school? (*multiple selection possible*)

	**Used in department**	**Permitted at dental school ***
Single-choice items (single-choice, best-answer, Type $A_{positive}$)	☐	
Single-choice items with **<u>negation in item stem</u>** (Type $A_{negative}$)	☐	
Multiple-choice items for which the number of answer options to be selected is **<u>not</u>** specified (multiple-select)	☐	
Multiple-choice items where the number of answer options to be selected is specified (PickN)	☐	
Alternate-choice items with exactly 2 answer options or true-false single-choice items	☐	
True-false multiple-choice items (Multiple-True-False, Kprim, Type K', Type X)	☐	
Extended matching items (Type R)	☐	
Providing a set of answer combinations which comprise secondary choices (Type K)	☐	
Other: ________________________	☐	

* according to your examination and program regulations, local examination guidelines, etc

7. To what percentage does each multiple-choice item type contribute to your department's item pool? / What is the maximum percentage of each individual item type that can be used within an examination at your dental school? (Please distribute 100 points across the listed item types)

	**Proportion among department's item pool**	**Requirement at dental school ***
Single-choice items (single-choice, best-answer, Type $A_{positive}$)		
Single-choice items with **<u>negation in item stem</u>** (Type $A_{negative}$)		
Multiple-choice items for which the number of answer options to be selected is **<u>not</u>** specified (multiple-select)		
Multiple-choice items where the number of answer options to be selected is specified (PickN)		
Alternate-choice items with exactly 2 answer options or true-false single-choice items		
True-false multiple-choice items (Multiple-True-False, Kprim, Type K', Type X)		
Extended matching items (Type R)		
Providing a set of answer combinations which comprise secondary choices (Type K)		
Other: ________________________		

* according to your examination and program regulations, local examination guidelines, etc

8. For each item type selected in question 6, please describe the scoring algorithm at the level of an individual item and provide any further requirements at your dental school *, if applicable.

	**Number of answer options or statements per item** *(eg, from 4 up to 6 statements)*	**Number of correct answer options or statements per item** *(eg, from 0 up to all statements)*	**Scoring** *(eg, 1 full credit point if all statements are marked correctly, ½ credit point if up to 1 statement is marked incorrectly, otherwise 0 credit points)*
Single-choice items (single-choice, best-answer, Type $A_{positive}$)			
Single-choice items with **negation in item stem** (Type $A_{negative}$)			
Multiple-choice items for which the number of answer options to be selected is **not** specified (multiple-select)			
Multiple-choice items where the number of answer options to be selected is specified (PickN)			
Alternate-choice items with exactly 2 answer options or true-false single-choice items			
True-false multiple-choice items (Multiple-True-False, Kprim, Type K', Type X)			
Extended matching items (Type R)			
Providing a set of answer combinations which comprise secondary choices (Type K)			
Other: ______________________			

* according to your examination and program regulations, local examination guidelines, etc

9. Do students in your department / at your dental school receive immediate feedback during an examination (eg, by using the answer-until-correct item type)?

	**Used in department**	**Permitted at dental school ***
Answer-until-correct testing method	☐	☐
Other: ______________________	☐	☐

* according to your examination and program regulations, local examination guidelines, etc

Please turn →

10. Are examination items in your department / at your dental school subjected to peer-review / a formal item review process?

	Used in department	Requirement at dental school *
Yes	☐	☐
No	☐	☐

* according to your examination and program regulations, local examination guidelines, etc

If "yes", how does the review process look like?

____

____

____

You are welcome to provide further information regarding the use of multiple-choice examinations in general or particularly regarding the scoring of multiple-choice items.

____

____

____

____

____

____

You are welcome to provide your contact details in case we have any questions. (voluntary)

____

____

____

Please provide us with a copy of your examination and program regulations / local examination guidelines regarding use and scoring of multiple-choice items at your dental school.

Thank you for your support and participation in our survey! Please use the provided and pre-paid return envelope. By returning the questionnaire, you give your informed consent for the anonymous evaluation of the provided answers.

Rössler et al: Use of Multiple-Choice Items in Summative Examinations: Questionnaire Survey Among German Undergraduate Dental Training Programs. ***JMIR Med Educ 2024; 10:e58126*. doi: 10.2196/58126**

Schematic overview of the item types mentioned in this survey

**Item type**	**Scheme**<br>*(round marking boxes represent 1 answer option to be selected (1 out of X), while square marking boxes imply that multiple answer options or statements (x out of X) can be chosen)*
Single-choice items (single-choice, best-answer, Type $A_{positive}$)	**Type $A_{positive}$**<br>Item stem<br>○ Answer option 1<br>○ Answer option 2<br>○ Answer option 3<br>○ ...<br>○ Answer option *n*
Single-choice items with **negation in item stem** (Type $A_{negative}$)	**Type $A_{negative}$**<br>Item stem with **negation**<br>○ Answer option 1<br>○ Answer option 2<br>○ Answer option 3<br>○ ...<br>○ Answer option *n*
Multiple-choice items for which the number of answer options to be selected is **not** specified (multiple-select)	**Conventional multiple-select**<br>Item stem<br>□ Answer option 1<br>□ Answer option 2<br>□ Answer option 3<br>□ ...<br>□ Answer option *n*
Multiple-choice items where the number of answer options to be selected is specified (PickN)	**PickN**<br>Item stem *(Please mark x options!)*<br>□ Answer option 1<br>□ Answer option 2<br>□ Answer option 3<br>□ ...<br>□ Answer option *n*
Alternate-choice items with exactly 2 answer options or true-false single-choice items	**Alternate-choice / true-false single-choice**<br>Statement true ○ false ○
True-false multiple-choice items (Multiple-True-False, Kprim, Type K', Type X)	**Multiple-True-False (Type K')**<br>True False<br>Statement 1 ○ ○<br>Statement 2 ○ ○<br>... ○ ○<br>Statement *n* ○ ○
Extended matching items (Type R)	**Extended matching (Type R)**<br>Answer options<br>A) Answer option 1<br>B) Answer option 2<br>C) Answer option 3<br>D) ...<br>...) Answer option *n*<br><br>Lead-in-statement<br>Item stem / situation 1 ___<br>Item stem / situation 2 ___<br>Item stem / situation 3 ___
Providing a set of answer combinations which comprise secondary choices (Type K)	**Type K**<br>A) Statement 1<br>B) Statement 2<br>C) Statement 3<br>D) ...<br>...) Statement *n*<br>*Please mark 1 answer option of the following secondary choices!*<br>○ Statements A and B are true<br>○ Only statement C is true<br>○ Only statement D is true<br>○ All statements are true<br>○ All statement are false
